# *Trypanosoma cruzi*: Time for International Recognition as a Foodborne Parasite

**DOI:** 10.1371/journal.pntd.0004656

**Published:** 2016-06-02

**Authors:** Lucy J. Robertson, Brecht Devleesschauwer, Belkisyolé Alarcón de Noya, Oscar Noya González, Paul R. Torgerson

**Affiliations:** 1 Parasitology Lab, Section for Microbiology, Immunology, and Parasitology, Department of Food Safety and Infection Biology, Norwegian University of Life Sciences, Adamstuen-Campus, Oslo, Norway; 2 Department of Virology, Parasitology and Immunology, Faculty of Veterinary Medicine, Ghent University, Ghent, Belgium; 3 Emerging Pathogens Institute and Department of Animal Sciences, University of Florida, Gainesville, Florida, United States of America; 4 Immunology Section, Instituto de Medicina Tropical, Facultad de Medicina, Universidad Central de Venezuela, Caracas, Venezuela; 5 Biohelmintiasis Section, Instituto de Medicina Tropical, Facultad de Medicina, Universidad Central de Venezuela, Caracas, Venezuela; 6 Centro para Estudios Sobre Malaria, Instituto de Altos Estudios “Dr. Arnoldo Gabaldón”, Instituto Nacional de Higiene, MPPS, Caracas, Venezuela; 7 Section of Epidemiology, Vetsuisse Faculty, University of Zürich, Zürich, Switzerland; Institute of Tropical Medicine, BELGIUM

## A Neglected Transmission Route of a Neglected Tropical Disease

Chagas disease is one of the “neglected tropical diseases” (NTDs) listed by the World Health Organization (WHO). Depending on the study, the global prevalence has been estimated to be between 9 and 10 million people, with a disease burden from 300,000 to over 800,000 disability adjusted life years (DALYs) [1–4]. At least 10,000 people die from Chagas disease annually. Chagas disease accounts for over 11% of heart failure in Brazil [3], and over 7% of deaths due to heart failure during 2006 were due to Chagas disease [5]. The global costs of Chagas disease have been estimated at US$7.19 billion per year.

Transmission of *Trypanosoma cruzi*, the protozoan etiological agent of Chagas disease, was traditionally considered almost exclusively vectorborne, transmitted by bugs (Hemiptera) in the family Reduviidae, sub-family Triatominae. However, other transmission routes exist, including intrauterine, by blood transfusions and organ transplantation, laboratory accidents, and foodborne transmission. In foodborne infections, food is, or more commonly, drinks are, contaminated with metacyclic trypomastigotes, which are then ingested by susceptible humans. These contaminant metacyclic trypomastigotes may be derived from either the feces of triatomines or from the whole insect. This indicates that although such transmission is not classical vector-borne transmission, the vector is still essential in this foodborne modality. Alternatively, contaminant metacyclic trypomastigotes have the potential to be derived from the secretions of reservoir hosts such as opossums that may also contaminate food [6]; for this transmission route the vector is not involved. It should be noted that as metacyclic trypomastigotes are inactivated by the action of drying or by low moisture content [7], drinks such as fruit juices are the most common transmission vehicles. Other food items may also be relatively unsuitable as transmission vehicles due to toxic effects, and some food preparation treatments may also inactivate the parasites. Whereas heating seems to inactivate trypomastigotes [8], experiments have indicated that they are quite resistant to refrigeration and freezing [9].

Whereas direct vectorborne transmission has gradually been controlled, particularly due to initiatives in housing organized by international health institutions such as WHO and PAHO [10], foodborne infection continues to be relatively neglected. Although several researchers have drawn attention to this mode of transmission (e.g. [11,12]), and foodborne Chagas disease was mentioned by the WHO Foodborne Disease Burden Epidemiology Reference Group (FERG), it was not considered of sufficient interest to merit evaluation with the resources available [13]. Similarly, a “call for action” article for Chagas disease [14] does not mention foodborne transmission.

## Why Is Foodborne *T*. *cruzi* Infection Becoming More Common?

Outbreaks of foodborne Chagas disease are documented from 1966 [15] and oral transmission may always have been the usual infection route between wild and domestic fauna; stercorarian transmission is relatively inefficient, and fur and thick skin create a barrier for cutaneous penetration [10,16]. Much of the experimental work on oral transmission using animal models was conducted between 1960 and 1980, and human outbreaks occur regularly. Furthermore, earlier clusters of cases previously considered vectorborne may have been foodborne [15]. However, although we can better recognize outbreaks of foodborne Chagas disease, there seems to be little doubt that foodborne transmission is increasingly reported. Since 2010, over 16 outbreaks of foodborne Chagas disease have been reported from at least four countries, involving around 170 individuals and a dozen deaths [15]. A summary of 73 reports from the past 10 years that includes 959 cases of acute Chagas disease suggests that 638 (>66%) were due to oral transmission, 258 congenital, and only 23 due to direct cutaneous vectorborne transmission [17]. The relative increase in foodborne transmission may not only reflect decreases in cutaneous vectorborne transmission due to efficient control of the vector *Triatoma infestans* by domiciliary spraying [18] but also result from alterations in aspects of ecology and host behavior [19]. Progressive invasion and domestication of wild triatomines has occurred in rural and urban localities. The species *Triatoma dimidiata*, *Panstrongylus rufotuberculatus*, *Rhodnius stali*, *Eratyrus mucronatus* and *Panstrongylus geniculatus* have all been reported as being domiciled in urban areas [18,20,21]. This could have exacerbated possibilities for foodborne transmission. As well as “urbanization” of wild triatomines and reservoir hosts, human encroachment into areas where wild reservoirs live may also exacerbate the potential for transmission, both orally and by direct vectorborne transmission, particularly as environmental imbalance caused by man through the invasion and deforestation of woodlands, results in reduction of the biodiversity of mammals available as food sources for triatomines [19]. In the Brazilian Amazon around 70% of cases of acute Chagas disease recorded between 2000 and 2010 were associated with food consumption [15]. Another important factor is that many more species of triatomines are suitable as vectors for oral transmission than cutaneous vectorborne transmission, as a rapid defecation reflex after feeding is unnecessary in foodborne transmission [15]. The rapidity of defecation following feeding has long been used as a measure of the suitability of different triatomine species for vectorial transmission, and a defecation index was even proposed as a measure of rapidity and frequency of defecations, and hence importance as a transmission agent following biting [22,23]. This means that triatomines such as *P*. *geniculatus*, the most widely distributed species in the Americas, are suitable for foodborne transmission, but not for cutaneous vectorborne transmission. In cities such as Caracas, the high prevalence of infection of *P*. *geniculatus* with *T*. *cruzi* [24,25], the wide distribution of this vector in neighborhoods, and its presence in homes during its immature stages (five nymph stages) are factors that lead to the direct contamination of food and beverages. Nymphs may easily contaminate food unnoticed due to their small size (1st nymphal instar is around 2 mm).

## Why Is Foodborne Transmission Potentially More Serious Than Vectorborne Transmission?

The route of infection influences the success of *T*. *cruzi* in its vertebrate host and also the severity of clinical outcome. Relevant factors include the number of metacyclic trypomastigotes, the biodeme involved, and the host’s immune response. The relatively high percentage of morbidity and mortality in the early stages of infection in foodborne transmission [26] have been related to higher parasite load and the efficient mechanism of infection through the stomach mucosa, as first demonstrated by Hoft et al. [27]. This is based on expression of gp82, a stage-specific surface glycoprotein that binds to gastric mucin and epithelial cells, triggering the signaling cascades leading to intracellular Ca^2+^ mobilization and promoting parasite entry [28]. During the early stages of infection, prolonged high fever occurs in 80–100% of cases with oral transmission, whereas with vectorborne transmission, symptoms are often mild or even absent [15]. Furthermore, cardiac pathology occurs relatively frequently, and is potentially severe. In the Chacao outbreak severe clinical signs occurred in 34.4% of patients, compared with 5–10% by cutaneous vectorborne transmission [29].

A range of experimental studies in mice has also demonstrated significantly greater infectivity through oral challenge [30,31]. As with many other unrelated foodborne parasites, the potential for heavy contamination of the infection vehicle is critical for transmission success. In vectorborne transmission, the parasite inoculum is through triatomine feces, perhaps containing 3,000–4,000 metacyclic trypomastigotes per μl, of which only a proportion succeed in penetrating the epidermis. However, *T*. *infestans* can harbor 684,000 infective trypomastigotes, able to infect hundreds by the oral route [15].

Timely diagnosis and effective treatment are important for decreasing disease progression and the likelihood of congenital transmission. However, the absence of the classical signs (a skin chagoma or Romaña's sign) and ignorance regarding the presence of triatomines may delay diagnosis in foodborne infections.

The importance of other routes of infection with *T*. *cruzi* should not be underestimated. These include the fact that transmission via blood transfusion and/or tissue donation can deliver high infectious inoculum and tends to result in the most virulent acute disease especially in immunosuppressed persons. Thus the greater likelihood of infecting higher numbers of people, combined with the potential for delayed diagnosis and more severe symptoms, means that foodborne Chagas disease is likely to have a greater impact than vectorborne transmission at both individual and community level. Nevertheless, it is important to remember that domestic vectors may still colonize houses across wide areas, and vector elimination efforts should be sustained [32].

## Estimating the Burden of Foodborne Chagas Disease

While the evidence of foodborne transmission of *T*. *cruzi* is convincing, to date there have been no systematic reviews or other published work that might indicate the proportion of Chagas disease transmitted via food. However, as an indication, data can be extracted from a narrative review of acute Chagas disease [17]. On a crude level, 638 of 959 cases (67%) were orally transmitted [17], and, in consideration of the large foodborne outbreaks that have been reported, we suggest that the majority of these would be through contaminated food, including beverages. Furthermore, 21 were through reactivation and 258 through congenital transmission. The former would have a primary transmission of similar proportions to the total, and likewise for women who transmit the parasite to their children congenitally. The only cases definitively not linked to oral transmission are the cutaneous vectorborne cases [12], and the transfusion and transplantation cases [5]. Adding in the unknown to this group, and making an adjustment (some reactivation and congenital cases would result from vectorborne transmission) would result in approximately 910 cases that are acquired through oral transmission (95%). Even if only around half of these are actually foodborne, this would result in 273,000 DALYs per annum attributable to contaminated food, assuming the Chagas disease burden reported in GBD 2010 [2]. This would rank foodborne Chagas disease as approximately the 8^th^ most important foodborne parasitic disease on a global basis [13], despite much of the world being free from this parasite. This may give some pointers as to the burden of foodborne Chagas disease, but it should be noted that both foodborne and vectorborne disease may also be sporadic and hence using outbreak data might underestimate the burden from vectorborne transmission. It should be noted that oral infection does not necessarily indicate contaminated food—*per os* infection can also occur from dirty hands contaminated with triatomine feces. Such an infection route is less likely to result in a large-scale outbreak. Furthermore, chronic disease, with no acute stage symptomatology, provides a substantial burden and the contribution of different transmission pathways leading to that clinical picture have yet to be addressed.

Clearly, a systematic review of the evidence rather than the superficial overview reported here might better untangle the contributions to the burden of Chagas disease by the different transmission pathways.

## Message to Stakeholders and Policy-Makers

WHO launched FERG in order to provide data and tools to support policy-makers and other stakeholders when setting appropriate, evidence-informed priorities of food safety at country level. However, by focusing on global impact, important pathogens that have a restricted distribution may be overlooked. It seems as though *T*. *cruzi* could be one of these. In calling to the relevant governments from Latin America and elsewhere for sustained support for prevention, control, and treatment of Chagas disease, Schmuñis [14] provided an overview of the disease, including transmission and mitigation initiatives; however, foodborne infection was not mentioned. Likewise, in the CODEX Alimentarius draft guidelines on the application of the general principles of food hygiene to the control of foodborne parasites, *T*. *cruzi* is not mentioned.

By concerted efforts, relevant authorities have managed to reduce vectorborne transmission of Chagas disease considerably. Foodborne transmission may be a more complex situation, with multiple and changing factors that mean transmission reduction may be more difficult to achieve. However, before we can think about control, the problem must be first acknowledged. The intention of this article is to bring foodborne transmission further onto the table.
